# Supplementary figures and images for: How do care home staff use non-pharmacological strategies to manage sleep disturbances in residents with dementia: The SIESTA qualitative study
Source: PLoS One. 2022 Aug 9;17(8):e0272814. doi: 10.1371/journal.pone.0272814 (PMC9362920; doi:10.1371/journal.pone.0272814)

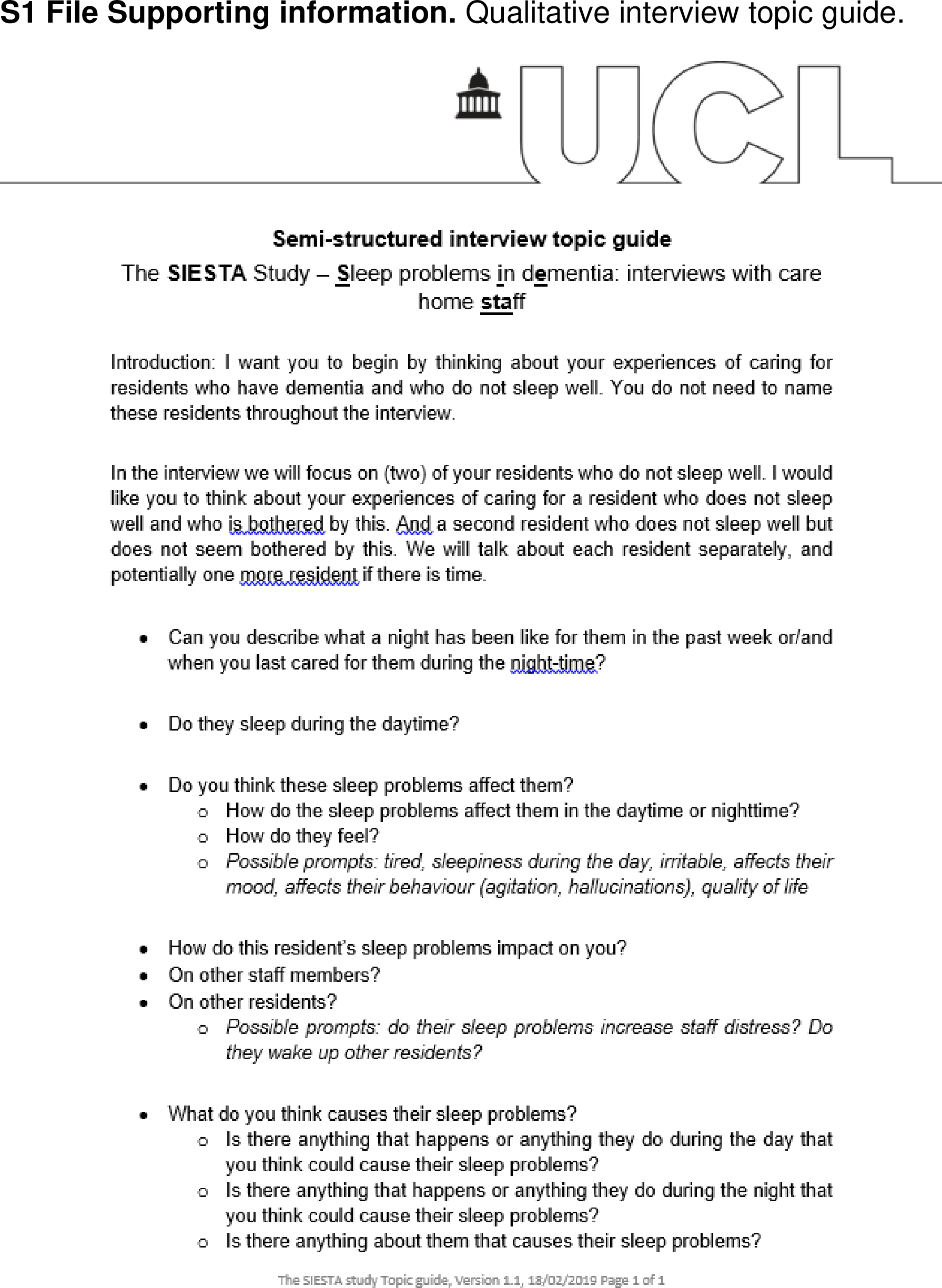

Supplement: S1 File — (TIF) [file pone.0272814.s001.tif]
