# Supplementary material for: How do care home staff use non-pharmacological strategies to manage sleep disturbances in residents with dementia: The SIESTA qualitative study
Source: PLoS One. 2022 Aug 9;17(8):e0272814. doi: 10.1371/journal.pone.0272814 (PMC9362920; doi:10.1371/journal.pone.0272814)
Supplement: Attachment — Submitted filename: PLOS ONE ms review December 2021.docx [file pone.0272814.s002.docx]

Manuscript review: Describing and overcoming barriers to home staff using non-pharmacological strategies for sleep disturbances in care home residents with dementia: the SIESTA qualitative study. Submitted to PLOS ONE. December 2021

Thank you for the opportunity to review this paper. According to the title, the focus is on (1) “describing and overcoming barriers” to the use of non-pharmacological strategies for sleep disturbances for (2) people with dementia in residential care. I comment on these two issues as they do not seem to be the focus of the paper. Information on “barriers to managing sleep disturbances” (Results) is not presented until page 12 (line 259) and appears minimal compared to the presented information on strategies. Further, on page 19 (line 414+) the authors write that “we did not check the diagnoses of the residents” so there is an assumption about residents having dementia that may be incorrect.

Financial disclosure – please correct the spelling/typographical error.

Page 1 (line 3): Suggest deleting the second mention of “care home”

Abstract (page 2)

Background – please correct the grammar in the initial sentences: “care homes” should be singular; “It is “should be “They are” (relating to sleep disturbances”

Methods: Clarify how many times you interviewed the staff.

Results: Information from “During the daytime ….” to “They used telecare to monitor night-time risk” seems to fit better in Methods. Given the title of the paper, I would expect data-based information about barriers to be presented in the Results.

Conclusions: The “range of techniques” mentioned here seems to fit better in Results. Line 40: “residents night-time needs” should be residents’ night-time needs

Page 3

Line 47: delete “base”

Line 48: Move “of hypnotics” to follow “randomised controlled trials; explain hypnotics

Line 55: Delete “however”

Line 59: Spell out NICE before you use the abbreviation

Line 62: Include further details about the evidence supporting multi-component approaches to sleep disturbances. Later, in your Results section, you talk about the strategies staff used. I need to understand these strategies and that understanding needs to come from the detailed information you present in your Introduction.

Line 64: End the sentence after “residents with dementia.” Next sentence could be “These factors include noise (17), light (18), other residents’ (correct the position of the apostrophe; and make this correction on page 4, line 68; and on page 15, line 318) wakefulness ….

Page 4

Line 75: End the sentence after “residents with dementia.” Delete mention of the interviews. Including this information suggests you have already published the results of the interviews in reference 3. If this is true, the current paper does not need to be published. Make sure your research question for the current paper is clearly stated.

Page 5

Line 93: Change “do” to “did”

Line 100: Include information on the length of the interviews and when they were completed

Line 104: Provide further details on “altered the guide iteratively”

Page 6

Line 110: Who transcribed the interviews? Need further details here. If the first author transcribed all the interviews, there is the risk of bias. You mention further down that two authors developed the thematic coding framework but I am confused about the sequence of events. Please explain what occurred when there was uncertainly or disagreement about coding.

Page 7

Line 122: Much of what is presented here seems to fit better in your earlier Methods section.

Line 132: Please describe your participants as well as refer to Table 1. I would like to know more about whether/how your 8+6 care staff were educated about evidence-based strategies.

Line 138: You need a transition here e.g., “Details are presented in the following sections” so that a reader knows details are forthcoming.

Line 139: Your subheading is “Daytime strategies” so on line 140, suggest deleting “To prevent night-time sleep disturbances”.

Page 9

Line 172: Change ‘do” to “go”

Page 15 - Discussion.

Line 308: Please address your research question - which, given your title, appears to be on addressing barriers – and explore the reasons for these. I feel that when you mention barriers, you are listing them and making general statements rather than delving into why they occurred and citing evidence to support how they might be overcome. I understand that your focus is on non-pharmacological strategies, but I think you need to address the drugs that residents have been prescribed. This is missing from the current paper and is relevant, particularly in relating to residents’ apparent fatigue and sleepiness during the day.
