# Supplementary material for: How do care home staff use non-pharmacological strategies to manage sleep disturbances in residents with dementia: The SIESTA qualitative study
Source: PLoS One. 2022 Aug 9;17(8):e0272814. doi: 10.1371/journal.pone.0272814 (PMC9362920; doi:10.1371/journal.pone.0272814)
Supplement: Attachment — Submitted filename: Response to reviewers July 2022.docx [file pone.0272814.s004.docx]

Dear PLOS ONE,

*RE: Manuscript review: How do care home staff use non-pharmacological strategies to manage sleep disturbances in residents with dementia: the SIESTA qualitative study*

Thank you for again reviewing our manuscript, and please find below our replies to the editors and reviewers comments, indicating where we have revised the manuscript as necessary.

Editors comments:

*Please review your reference list to ensure that it is complete and correct. If you have cited papers that have been retracted, please include the rationale for doing so in the manuscript text, or remove these references and replace them with relevant current references. Any changes to the reference list should be mentioned in the rebuttal letter that accompanies your revised manuscript. If you need to cite a retracted article, indicate the article’s retracted status in the References list and also include a citation and full reference for the retraction notice.*

We have now reviewed the reference list and found that no references were redacted and have sure references are as complete as possible. We updated the reference for a Cochrane review (McCleery, 2016) to a more recently updated version of this review (McCleery, 2020). We have also added in a few additional references in the data analysis section, and the strengths and weaknesses section.

Reviewers' comments:

*4. Have the authors made all data underlying the findings in their manuscript fully available?

The* [*PLOS Data policy*](http://www.plosone.org/static/policies.action#sharing) *requires authors to make all data underlying the findings described in their manuscript fully available without restriction, with rare exception (please refer to the Data Availability Statement in the manuscript PDF file). The data should be provided as part of the manuscript or its supporting information, or deposited to a public repository. For example, in addition to summary statistics, the data points behind means, medians and variance measures should be available. If there are restrictions on publicly sharing data—e.g. participant privacy or use of data from a third party—those must be specified.*

*Reviewer #1: No*

*Reviewer #2: Yes*

*Reviewer #3: Yes*

All data have been made available and this was specified in the response to reviewers after the first review.

*6. Review Comments to the Author

Please use the space provided to explain your answers to the questions above. You may also include additional comments for the author, including concerns about dual publication, research ethics, or publication ethics. (Please upload your review as an attachment if it exceeds 20,000 characters)*

*Reviewer #1: The authors have adequately addressed my concerns. Good study albeit not being very innovative. The manuscript can be published in its present form*

*Reviewer #2: A thoughtful revision - my concerns have been largely addressed. The Paper will be of interest in the care sector.*

*Reviewer #3: Thank you for your careful attention to my earlier comments and suggestions.
I have a few more for clarity and grammar:
- Please check your use of "residents" and make the needed correction when you mean the possessive form, i.e. residents'*

This has now been checked & edited where necessary.

*- On page 10, line 193: please change the heading to "Evening strategies to promote sleep" This then matches the earlier heading of "Daytime strategies..." I ask you to delete "good" as you have not measured the quality of sleep.*

This has now been changed.

*-On page 11, line 210: please change the heading to "Night-time strategies to manage sleep".*

This has now been changed.

*- Page 15, line 296: Change "these" to "sleep"*

This has now been changed.

*-Page 20, line 409: Change "is" to "are" (but there are often...)*

This has now been changed.

*- Page 21, line 431: Change "females" to "female"*

This has now been changed.

Yours sincerely,

Lucy Webster

(On behalf of all the authors)
